# Tunneling nanotube formation promotes survival against 5‐fluorouracil in MCF‐7 breast cancer cells

**DOI:** 10.1002/2211-5463.13324

**Published:** 2021-11-17

**Authors:** Kaylyn Kato, Kim Tho Nguyen, Carl W. Decker, Kai H. Silkwood, Sydney M. Eck, Jeniffer B. Hernandez, Jerome Garcia, Derick Han

**Affiliations:** ^1^ School of Pharmacy and Health Sciences Keck Graduate Institute Claremont CA USA; ^2^ Department of Biology University of LaVerne CA USA

**Keywords:** 5‐fluorouracil, chemotherapy, cytochalasin B, doxorubicin, drug resistance, tunneling nanotubes

## Abstract

Tunneling nanotubes (TNTs) are F‐actin‐based open‐ended tubular extensions that form following stresses, such as nutritional deprivation and oxidative stress. The chemotherapy agent 5‐fluorouracil (5‐FU) represents a significant stressor to cancer cells and induces thymidine deficiency, a state similar to nutritional deprivation. However, the ability of 5‐FU to induce TNT formation in cancer cells and potentially enhance survival has not been explored. In this study, we examined whether 5‐FU can induce TNT formation in MCF‐7 breast cancer cells. Cytotoxic doses of 5‐FU (150–350 μm) were observed to significantly induce TNT formation beginning at 24 h after exposure. TNTs formed following 5‐FU treatment probably originated as extensions of gap junctions as MCF‐7 cells detach from cell clusters. TNTs act as conduits for exchange of cellular components and we observed mitochondrial exchange through TNTs following 5‐FU treatment. 5‐FU‐induced TNT formation was inhibited by over 80% following treatment with the F‐actin‐depolymerizing agent, cytochalasin B (cytoB). The inhibition of TNTs by cytoB corresponded with increased 5‐FU‐induced cytotoxicity by 30–62% starting at 48 h, suggesting TNT formation aides in MCF‐7 cell survival against 5‐FU. Two other widely used chemotherapy agents, docetaxel and doxorubicin induced TNT formation at much lower levels than 5‐FU. Our work suggests that the therapeutic targeting of TNTs may increase 5‐FU chemotherapy efficacy and decrease drug resistance in cancer cells, and these findings merits further investigation.

## Graphical abstract

Tunneling nanotubes (TNTs) are F‐actin–based tubular extensions that form following stress. Cytotoxic doses of 5‐fluorouracil (5‐FU) significantly induced TNT formation in MCF‐7 cells, which acted as conduits for mitochondrial exchange. The inhibition of TNTs by cytochalasin B increased 5‐FU cytotoxicity suggesting that the targeting of TNTs may increase 5‐FU efficacy in cancer cells.

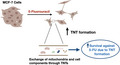

**Abbreviations**
5‐FU: 5‐fluorouracil
CytoB: cytochalasin B
DOX: doxorubicin
DTX: docetaxel
MDR1: multidrug resistance protein 1
TNTs: tunneling nanotubes
TS: thymidylate synthase

Tunneling nanotubes (TNTs) are F‐actin based open‐ended tubular extensions that are utilized for cell‐cell communication [1, 2, 3]. The formation of TNTs has been observed in a wide range of cells including fibroblasts, epithelial cells, neurons, and numerous transformed cells. While TNTs may exist at low levels under basal conditions in most cells, their formation is enhanced in response to various stresses including nutritional deprivation, UV light, oxidative stress, and cytotoxic agents [3, 4, 5]. TNTs formed during stress become conduits for exchange of proteins and organelles, such as mitochondria, that may aide in cell survival [6]. For example, the exchange of mitochondria through TNTs was shown to rescue PC12 cells that were injured by UV light [4]. TNTs have also been shown to be conduits for transfer of pathogens such as viruses (retroviruses, herpesviruses, orthomyxoviruses) and prions [7].

While TNT formation has been observed in many different cell types, it may become upregulated with malignant transformation, as TNTs have been observed at elevated levels in tumor cells including mesothelioma, glioblastoma, and pancreatic carcinomas [5, 8, 9]. In pancreatic cancer cells, TNTs were observed to facilitate the intercellular redistribution of doxorubicin (DOX), which allowed for greater resistance to the drug [9]. Cancer cells have also been shown to use TNTs to transfer protein involved in drug resistance such as the multidrug resistance protein 1 (MDR1) [10, 11]. Consequently, TNTs represent potential therapeutic targets to enhance chemotherapy efficacy and prevent drug resistance in cancer cells.

5‐Fluorouracil (5‐FU) is a widely utilized chemotherapy agent used in the treatment of a broad range of cancers including breast, colorectal, stomach, and pancreatic cancers [12]. 5‐FU is a pro‐drug that is metabolized to various products with anti‐cancer properties, including fluorodeoxyuridine monophosphate (FdUMP) which inhibits thymidylate synthase (TS), an enzyme essential in the *de novo* synthesis of thymidine [13]. Consequently, the cytotoxic mechanism of 5‐FU may be due in part to a deficiency in thymidine, which is needed for DNA synthesis. Like all chemotherapy drugs, cancer cells acquire resistance to 5‐FU through various mechanisms including amplification of TS, mutation of TS, and upregulation of MDR1 [14, 15].

5‐FU treatment to cancer cells represents a significant stressor to cancer cells and induces thymidine deficiency, a state similar to nutritional deprivation, which are known to increase TNT formation. However, the ability of 5‐FU to induce TNT formation in cancer cells and potentially enhance survival has not been explored. In this work, we examine these questions regarding 5‐FU and TNTs in MCF‐7 breast cancer cells.

## Materials and methods

### Materials

MCF‐7 breast cancer cells were obtained from ATCC (Manassas, VA, USA) and cultured using their established guidelines. All culturing reagents were also obtained from ATCC. All chemicals including 5‐FU, docetaxel (DTX), DOX, paraformaldehyde, and cytochalasin B (cytoB) were obtained from Sigma‐Aldrich (St. Louis, MO, USA). The fluorescent dyes phalloidin rhodamine and MitoTracker Green were obtained from Thermo‐Fisher (Waltham, MA, USA).

### Chemotherapy treatment to MCF‐7 cells and determination of cell viability

MCF‐7 cells were grown to ~ 70–80% confluence and then treated with various chemotherapy drugs listed above. 5‐FU was dissolved in DMSO for cell treatment. All control cells received equal doses of DMSO as vehicle control. Every 24 h media was changed and fresh chemotherapy agents were given to MCF‐7 cells. In experiments involving cytoB, cells were treated with cytoB 1 h prior to 5‐FU treatment. Cell viability was determined by trypan blue. The number of viable attached cells were counted using image j software (U. S. National Institutes of Health, Bethesda, MD, USA). Control cells or cells treated with cytoB alone generally reached confluence approaching greater than 95% following 48 h, thus TNT counts and cell viability were no longer determined past this time point. Statistical analysis was performed using Student’s *t*‐tests or ANOVA, with a significance *P*‐value of 0.05 or less.

### Quantification of TNT formation in MCF‐7 cells

A Nikon TE2000 inverted microscope (Nikon Instruments Inc., Melville, NY, USA) with 10× objective lens was used to count the number of TNTs in a minimum of 10–20 randomly chosen fields at various time points. A minimum of 700 cell were counted. TNTs were identified and counted using morphologic criteria previously described [5, 8, 9]: (a) TNTs were not adherent to the substratum of tissue culture plates, (b) the width of TNTs were 1000 nm or less, and (c) the site of TNT extrusion from the plasma membrane were narrow. Other cellular extensions such as filopodia, were observed with drug treatment but not counted as TNTs due to their failure to meet these criteria.

### Staining MCF‐7 cells with fluorescent dyes

To prepare cells for fluorescent staining, cells were cultured in one‐ or two‐well sterile tissue culture‐treated chamber slides (Lab‐Tek II Chamber Slides; Nunc, Rochester, NY, USA) or on sterile poly‐l‐lysine (1 mg·mL^−1^; Sigma‐Aldrich) coated glass coverslips for 48–72 h. Following treatment with the drugs described above, cells were fixed to preserve TNTs with 16% w/v paraformaldehyde. Fluorescent staining for actin was performed with phalloidin rhodamine (4 units·mL^−1^) in culture medium with 0.05% Triton X. Mitochondria were stained using MitoTracker Green (100 nm) in media. Images were captured using a Leica SP5 Confocal Microscope with metamorph software (Leica Microsystems, Bannockburn, IL, USA).

## Results

### 5‐FU treatment induces TNT formation in MCF‐7 cells

**Fig. 1:**
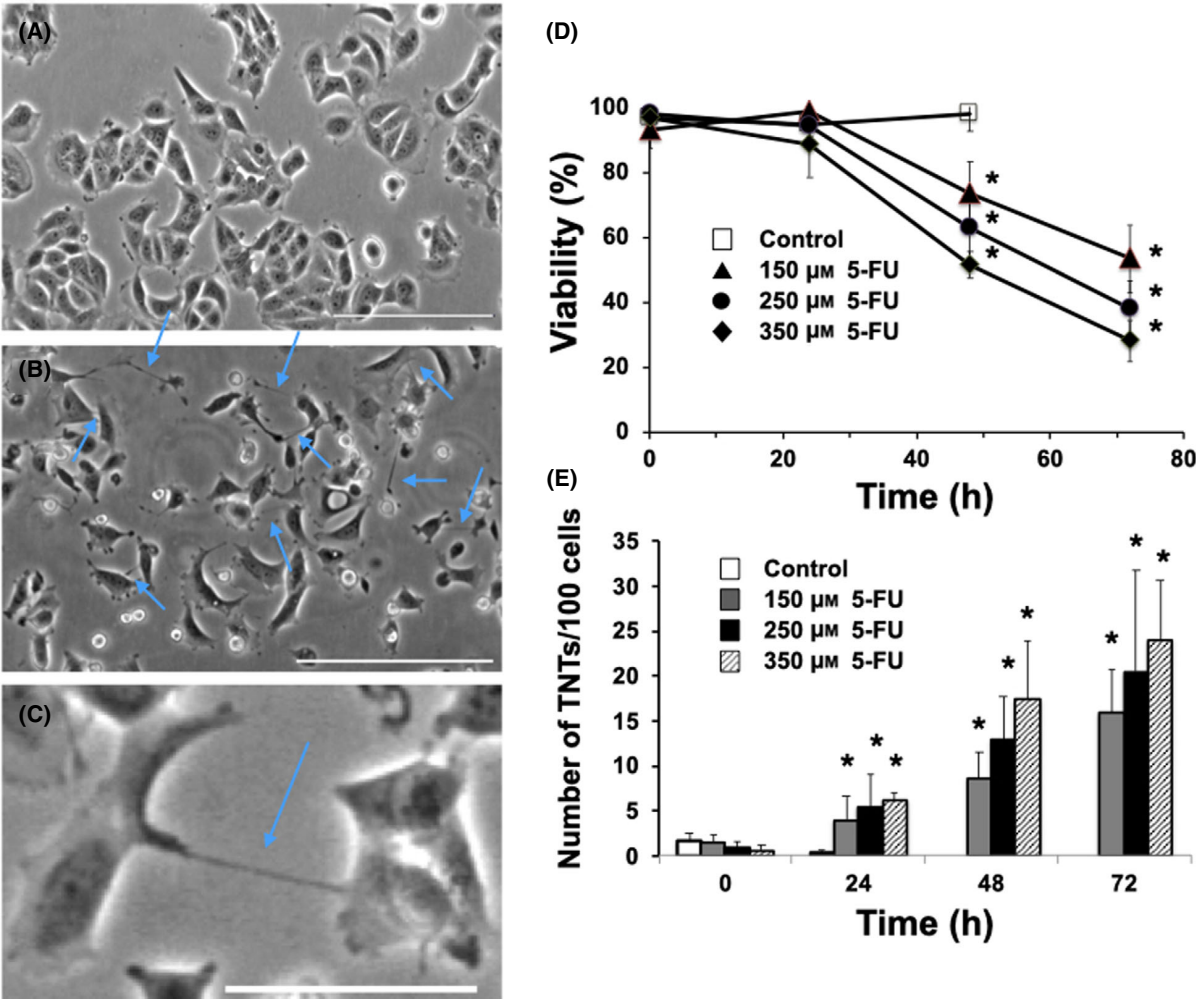
Cytotoxic doses of 5‐FU treatment induces TNT formation in MCF‐7 cells. (A) Control MCF‐7 cells. (B) MCF‐7 cells treated with 150 μm 5‐FU at 48 h. Blue arrows point to TNT structures formed following 5‐FU treatment. Scale bar = 200 μm. (C) Close up image of MCF‐7 cells treated with 150 μm 5‐FU at 24 h. Scale bar = 50 μm. (D) Time course of MCF‐7 viability following treatment with various doses of 5‐FU: 150 μm (▲), 250 μm (●), and 350 μm (♦) and vehicle control (□). (E) Time course of TNT formation following 5‐FU treatment in MCF‐7 cells. MCF‐7 cells following 5‐FU treatment: 150 μm (grey bar), 250 μm (black bar), 350 μm (striped bar) and vehicle control (white bar). MCF‐7 cells were brought to 70–80% confluence and treated various doses with 5‐FU. Viability was measured by trypan blue in both attached and detached cells. TNT was counted based on the criteria outlined in the Materials and methods. *N* = 4 experiments. Error bars = standard deviation. *P < 0.05 compared to control at 0 time following ANOVA analysis.

MCF‐7 breast cancers cells aggregate into clusters under basal conditions (Fig. 1A). The treatment of MCF‐7 cells with cytotoxic doses of 5‐FU (150–350 μm) resulted in a significant decline in clustering, as dead cells (identified by trypan blue staining) generally detached and became floating. As MCF‐7 cells became less clustered, a significant increase in TNTs formation to maintain cell‐cell contact occurred (Fig. 1B,C). All cytotoxicity doses of 5‐FU tested significantly increased TNT formation (Fig. 1D,E), while non‐cytotoxic doses of 5‐FU (100 μm and lower) did not significantly increase TNT formation (data not shown). No significant differences in TNT formation were observed between the cytotoxic doses of 5‐FU examined. TNT formation was most prominent at 48–72 h following 5‐FU treatment. The nanotubes observed following 5‐FU treatment were thin and sometimes out of the standard focal plane, the standard morphologic characteristics of TNTs [5, 8, 9]. TNT formation generally occurred between cells, but in some cases formed between cells and microplasts (migrating viable cytoplasmic fragments), which has been previously observed [16].

**Fig. 2:**
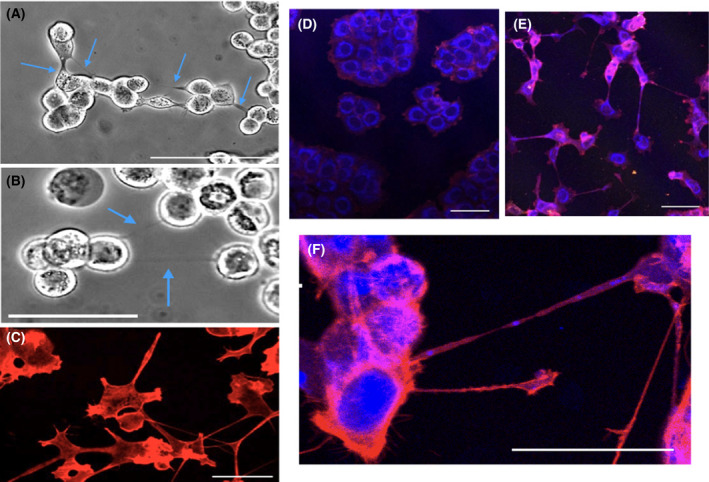
Characterization of TNTs formed in MCF‐7 cells following 5‐FU treatment. Key characteristics of TNTs, resistance to trypsin digestion and staining for F‐actin, are shown. (A) MCF‐7 cells (250 μm 5‐FU at 48 h) treated with trypsin (30 min) without disruption of TNTs. Scale bar = 200 μm. (B) MCF‐7 cells (150 μm 5‐FU at 48 h) treatment (60 min) with trypsin does not disrupt TNTs. Scale bar = 100 μm. (C) MCF‐7 cells (250 μm 5‐FU at 48 h) stained with the F‐actin dye, rhodamine phalloidin. Scale bar = 25 μm. Transport of mitochondria through TNTs formed following 5‐FU treatment to MCF‐7. MCF‐7 cells were treated with 5‐FU or vehicle control for 48 h and fixed. TNTs were stained with rhodamine phalloidin (F‐actin dye), while mitochondria were stained using MitoTracker Green. (D) Control MCF‐7 cells. Scale bar = 50 μm. (E) MCF‐7 cells treated with 250 μm 5‐FU at 48 h. (F) Close up of MCF‐7 cells treated with 250 μm 5‐FU at 48 h. Scale bar = 50 μm.

We next performed measurements to confirm that the nanotube structures formed following 5‐FU treatment had the morphologic features of TNTs and not filopodia or other similar structures. TNTs are resistant to trypsin digestion [1, 8], and similarly we observed the nanotubes formed by 5‐FU treatment were resistant to trypsinization (30–60 min; Fig. 2A,B). TNTs are primarily composed of F‐actin [1, 2, 3], and the tubular structures stained with the F‐action dye, rhodamine phalloidin (Fig. 2C). Finally, TNTs but not filopodia are conduits for exchange of cellular materials including ions, proteins, and organelles such as mitochondria [17]. Our work similarly shows mitochondria were transported between cells along TNTs following 5‐FU treatment (Fig. 2D–F). In particular, Fig. 2F clearly shows that mitochondria, stained with Mitotracker green, localized in TNTs, stained with rhodamine phalloidin. A large number of TNTs contained mitochondria at various points, suggesting mitochondrial movement between cells through TNTs occurred.

### TNT formation enhances MCF‐7 cell survival against 5‐FU treatment

**Fig. 3:**
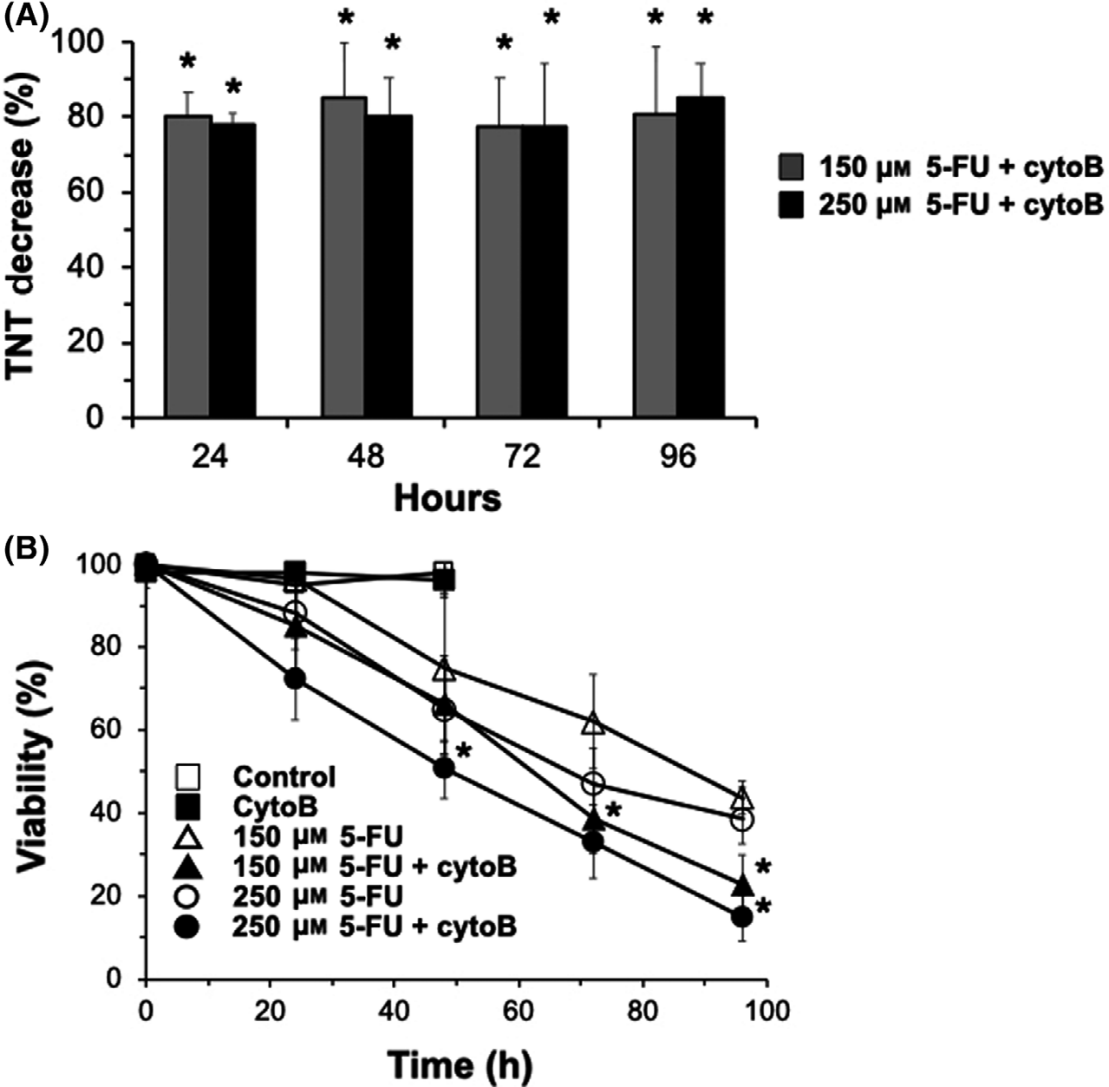
CytoB treatment inhibits TNT formation and enhances 5‐FU cytotoxicity in MCF‐7 cells. MCF‐7 cells were pretreated with cytoB for 1 h prior to 5‐FU treatment. (A) Inhibition of 5‐FU‐induced TNT formation by cytoB treatment. 5‐FU 150 μm (grey bar) and 5‐FU 250 μm (black bar) pretreated with cytoB (500 nm). (B) Time course of MCF‐7 viability following 5‐FU treatment in the presence and absence of cytoB. 5‐FU 150 μm (▵), 5‐FU 250 μm (○), vehicle control (□), 5‐FU 150 μm + cytoB (▲), 5‐FU 250 μm + cytoB (●), cytoB (■). Viability was measured by trypan blue in both attached and detached cells. TNT was counted based on the criteria outlined in the Materials and methods. *N* = 4 experiments. Error bars = standard deviation. *P < 0.05 compared to equivalent 5‐FU samples without cytoB treatment using *T*‐test.

We subsequently examined whether TNTs play a role in survival following 5‐FU treatment, by inhibiting TNT formation using cytochalasin B (cytoB), an F‐actin‐depolymerizing agent [4, 18]. Little cytotoxicity was observed with cytoB treatment alone even at high doses (< 2 μm) in MCF‐7 cells (data not shown). We found 500 nm of cytoB inhibited 5‐FU induced‐TNT formation by greater than 80% (Fig. 3A). The inhibition of TNTs by cytoB corresponded with increased 5‐FU induced cytotoxicity by 30–62% at 48–96 h (Fig. 3B). The enhanced cytotoxicity of 5‐FU with cytoB treatment was observed with both doses of 5‐FU (150 and 250 μm), suggesting TNT formation following 5‐FU treatment helps improve survival in MCF‐7 cells.

### TNT formation in MCF‐7 cells following various chemotherapy treatments

**Fig. 4:**
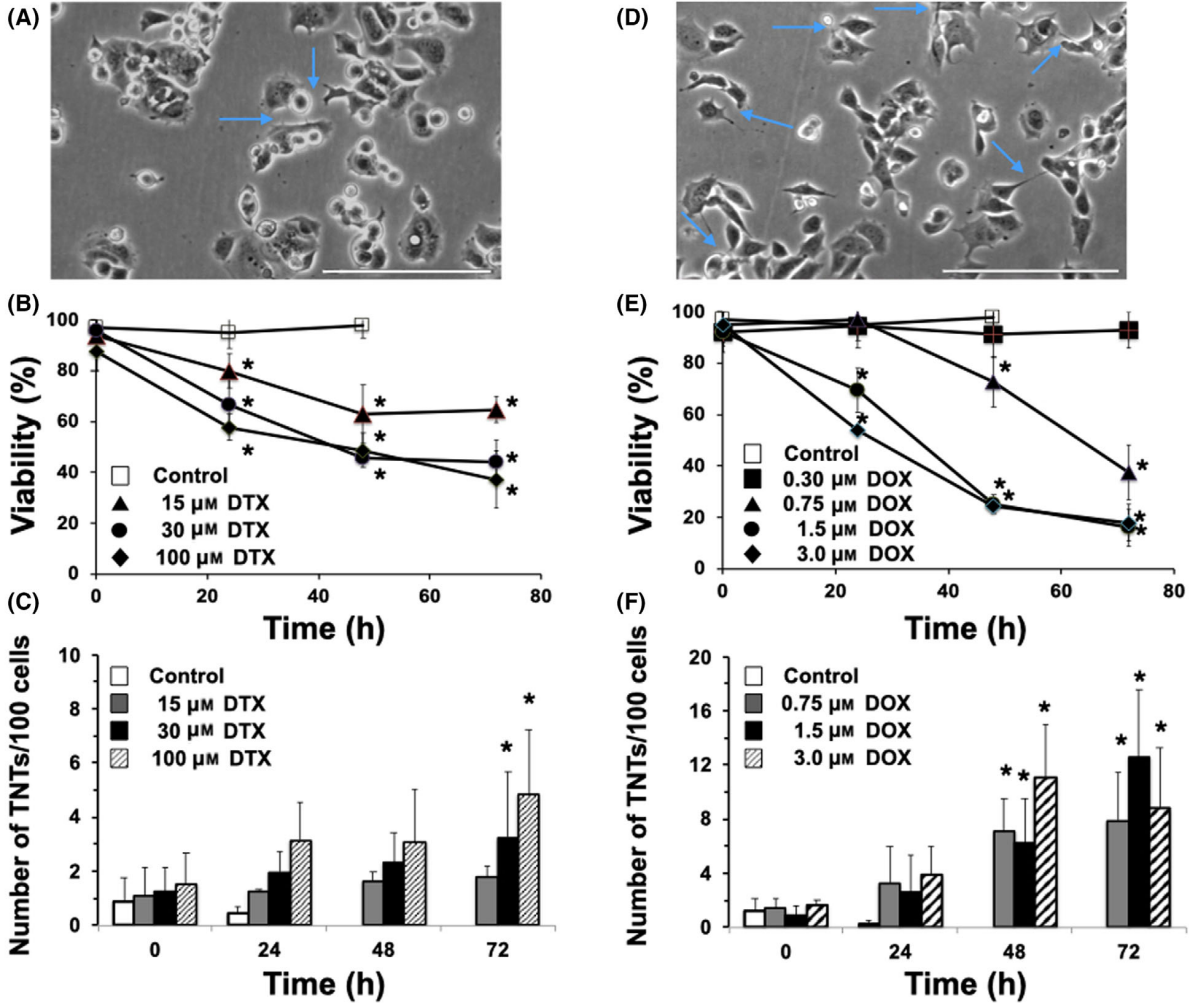
Effect of cytotoxic doses of DTX and DOX treatment on TNT formation in MCF‐7 cells. (A) MCF‐7 cells treated with 30 μm DTX at 72 h. Blue arrows point to some TNT structures formed following DTX treatment. (B) Time course of MCF‐7 viability following treatment with various doses of DTX: 15 μm (▲), 30 μm (●), and 100 μm (♦) and vehicle control (□). (C) Time course of TNT formation in MCF‐7 cells following DTX treatment. DTX 15 μm (grey bar), DTX 30 μm (black bar), and DTX 100 μm (striped bar) μm and vehicle control (white bar). (D) MCF‐7 cells treated with 1.5 μm DOX at 48 h. Blue arrows point to some TNT structures formed following DOX treatment. (E) Time course of MCF‐7 viability following treatment with various doses of DOX: 0.30 μm (■), 0.75 μm (▲), 1.5 μm (●), and 3 μm (♦) and vehicle control (□). (F) Time course of TNT formation following DOX treatment in MCF‐7 cells. DOX 0.75 μm (grey bar), DOX 1.5 μm (black bar), DOX 3 μm (striped bar) μm and vehicle control (white bar). MCF‐7 cells were brought to 70–80% confluence and treated various doses with DOX. Viability was measured by trypan blue in both attached and detached cells. TNTs were counted based on the criteria outlined in the Materials and methods. Scale bar = 200 μm. *N* = 4 experiments. Error bars = standard deviation. *P < 0.05 compared to control at 0 time following ANOVA analysis.

Finally, we examined if TNT formation is unique to 5‐FU treatment or if it is a general consequence of cytotoxic drugs by treating MCF‐7 cancer cells with other chemotherapy agents. DTX and DOX are commonly used chemotherapy agents with very different cytotoxic mechanisms than 5‐FU. We tested these drugs for their capacity to generate TNTs in MCF‐7 cells. DTX, a chemotherapy agent that inhibits microtubular depolymerization and induces bcl‐2 phosphorylation [19], was found to be cytotoxic to MCF‐7 cells (Fig. 4A,B), although a strict dose‐cytotoxic curve was not observed (Fig. 4B). DTX treatment inhibited cell growth as cell cycle arrest was likely induced. Only low levels of TNTs formed following cytotoxic doses of DTX, generally ~ 5–6 fold lower than observed with 5‐FU (Fig. 4C). MCF‐7 cells appear to still remain clustered even following DTX‐induced death (Fig. 4A), which may underlie a decrease TNT formation compared to 5‐FU.

Doxorubicin is an anthracycline chemotherapy agent associated with many cytotoxic mechanisms, including generation of reactive oxygen species, intercalation of DNA, and inhibition of the ligase function of topoisomerase‐II [20]. DOX was very cytotoxic to MCF‐7 cells, although a strict dose dependent cytotoxic relationship was not observed (Fig. 4E). 0.30 μm DOX caused no death, while 0.75 μm caused significant death in MCF‐7 cells. DOX treatment also caused TNT formation (Fig. 4D,F), at levels greater those observed with DTX, but less than 5‐FU at all doses examined (~ 2 fold lower than 5‐FU). DOX, like DTX, appeared more clustered (Fig. 4D) than 5‐FU at cytotoxic doses examined.

## Discussion

In this work, we demonstrated that 5‐FU was particularly effective in inducing TNT formation in comparison with other chemotherapy drugs, DOX and DTX. It was also notable that DTX, which primarily kills cancer cells by inhibiting microtubular depolymerization, was the least effective drug tested in causing TNT formation. DTX is not associated with stresses known to cause TNT formation, such as increased reactive oxygen species generation [19], which DOX induces in cells, or nutritional deprivation, which 5‐FU may mimic by depleting thymidine through inhibition of TS. To further understand why 5‐FU was so potent in inducing TNT formation in MCF‐7 cells, a detailed examination of signaling pathways regulating TNT formation will need to be done. The regulation of TNT formation is complex and has been shown to be modulated by various signaling pathways including GTPases Rab11a and Rab8a, Wnt/Ca2+ pathways, p53, NF‐κB pathway, and the Akt–PI3K–mTor pathway in various cells [2, 3, 21, 22, 23]. It is also possible that TNT formation may be a consequence of stress‐induced detachment of cellular clusters. Two major models of TNT formation have been proposed: (a) TNT formation from filopodium‐like protrusions that attach to another cell, and (b) TNT originate from gap junctions from two attached cells that become separated [2, 3]. Given that MCF‐7 cells are highly attached in clusters, it is likely the latter model may be more predominant in TNT formation in MCF‐7 cells following chemotherapy treatment. 5‐FU may have induced greater TNT formation due to greater cellular detachment of gap junctions that extend to become TNTs, which needs further exploration. Since 5‐FU is primarily cytotoxic to cells in the S phase, it likely that cells in the S phase are the first to die following 5‐FU treatment, which breaks up MCF‐7 cell clusters and helps initiate TNT formation in the cells that survive.

CytoB, a mycotoxin that inhibits F‐actin polymerization, had been previously demonstrated to inhibit TNT formation in various cells [4, 18]. Similarly, in MCF‐7 cells, we observed cytoB could effectively inhibit TNT formation by greater than 80%, which corresponded with enhanced 5‐FU cytotoxicity by 30–62%. The potentiation of 5‐FU cytotoxicity by cytoB suggests that TNT formation is aiding cell survival in MCF‐7 cells. In PC12 cells, the transfer of mitochondria through TNTs was found to rescue UV‐damaged cells from apoptosis [4]. While in pancreatic cancer cells, TNTs were used to redistribute DOX between cells to enhance survival [9]. In MCF‐7 cells, TNT formation could aide in survival through redistribution of 5‐FU, mitochondria transfer, or transfer of cellular components such as TS or thymidine. CytoB broadly effects F‐actin polymerization, and thus it is possible cytoB enhanced 5‐FU cytotoxicity may have occurred through inhibition of F‐actin dependent pathways independent of TNTs. However, our data suggests that there was a strong correlation between a decrease in TNT levels and an increase 5‐FU cytotoxicity. Also in our studies, cytoB‐induced inhibition of TNT formation was lower than observed in some other studies, wherein cytoB could inhibit TNT formation by greater than 95% [4]. While TNTs are primarily composed of F‐actin, microtubules or cytokeratin filaments have been detected in TNTs in various cells [24]. Thus, lower efficacy in cytoB inhibiting TNTs in MCF‐7 cells may suggest other filaments help construct TNTs when F‐actin is inhibited by cytoB in MCF‐7 cells.

Tunneling nanotube formation has been observed in a wide range of cancer cells including solid tumors [8]. TNTs may not only aide in the survival of cancer cells but are also likely to promote drug resistance [9, 10, 11]. Chemotherapy agents such as 5‐FU utilized in combination chemotherapy are initially very effective in treating metastatic breast cancer, but lose effectiveness overtime as cancer cells develop drug resistance. Drug resistance to 5‐FU involves many mechanisms including mutations in the TS and upregulation of drug efflux transporters (i.e. MDR1) [14, 15]. MDR1 has been shown to be transferred between cells through TNTs, and thus could also be a mechanism by which TNTs promote cell survival in MCF‐7 cells following 5‐FU treatment [10, 11]. It is likely that TNTs can transport other proteins such as TS to promote survival and drug resistance in cancer cells following chemotherapy treatment. Taken together, the therapeutic targeting of TNTs may increase 5‐FU efficacy and decrease drug resistance in cancer cells and merits further investigation.

## Conflict of interest

The authors declare no conflict of interest.

## Author contributions

KK, KTN, CWD, SME and KHS carried out the experiments in paper and help edit the manuscript. JBH and JG helped in experimental design and editing the manuscript. DH oversaw the project and wrote the manuscript.

## Data Availability

Data available on request from the authors.
